# A nomogram based on clinicopathological features and serological indicators predicting breast pathologic complete response of neoadjuvant chemotherapy in breast cancer

**DOI:** 10.1038/s41598-021-91049-x

**Published:** 2021-05-31

**Authors:** Yijun Li, Jian Zhang, Bin Wang, Huimin Zhang, Jianjun He, Ke Wang

**Affiliations:** Department of Breast Surgery, First Affiliate Hospital, Xi’an Jiaotong University, 277 Yanta West Road, Xi’an, 710061 People’s Republic of China

**Keywords:** Breast cancer, Cancer epidemiology, Surgical oncology, Breast cancer, Predictive markers

## Abstract

A single tumor marker is not enough to predict the breast pathologic complete response (bpCR) after neoadjuvant chemotherapy (NAC) in breast cancer patients. We aimed to establish a nomogram based on multiple clinicopathological features and routine serological indicators to predict bpCR after NAC in breast cancer patients. Data on clinical factors and laboratory indices of 130 breast cancer patients who underwent NAC and surgery in First Affiliated Hospital of Xi'an Jiaotong University from July 2017 to July 2019 were collected. Multivariable logistic regression analysis identified 11 independent indicators: body mass index, carbohydrate antigen 125, total protein, blood urea nitrogen, cystatin C, serum potassium, serum phosphorus, platelet distribution width, activated partial thromboplastin time, thrombin time, and hepatitis B surface antibodies. The nomogram was established based on these indicators. The 1000 bootstrap resampling internal verification calibration curve and the GiViTI calibration belt showed that the model was well calibrated. The Brier score of 0.095 indicated that the nomogram had a high accuracy. The area under the curve (AUC) of receiver operating characteristic (ROC) curve was 0.941 (95% confidence interval: 0.900–0.982) showed good discrimination of the model. In conclusion, this nomogram showed high accuracy and specificity and did not increase the economic burden of patients, thereby having a high clinical application value.

## Introduction

With the continuous progression and development of science and technology, scientists' understanding of breast cancer is also deepening. Precision treatment in breast cancer is now an option for many patients. Compared with traditional postoperative adjuvant chemotherapy, preoperative neoadjuvant chemotherapy (NAC) is increasingly used in the treatment of locally advanced breast cancer^1^ and offers the following advantages: (1) for patients with large primary tumors or more axillary lymph node metastasis, NAC can reduce the size of both the tumor and metastatic lymph nodes, thereby offering a chance of surgery to inoperable patients and reducing the incidence of axillary lymph node biopsy (ALNB); and (2) in addition, for the patients with large tumors and breast-conserving intention, NAC can improve the success rate of breast-conserving surgery (BCS) and possibly make the preserved breast appear more aesthetic^2^. At present, the biggest debate regarding NAC vs. postoperative adjuvant chemotherapy in breast cancer is whether the former can further improve prognosis. Most of the current clinical studies have not confirmed this in the whole population^3–5^. However, it is encouraging that almost all studies have confirmed that pathological complete response (pCR) after NAC can predict long-term survival in some specific subtypes of breast cancer, such as HER2-positive and triple negative breast cancer^6,7^. To some extent, pCR can be used as a surrogate marker for disease-free survival (DFS) and overall survival (OS)^8–10^. Therefore, the prediction of pCR before the start of NAC is very important for clinicians to formulate treatment plans for patients. The treatment plan should be altered by immediately discontinuing NAC in favor of surgical treatment for patients with a lower probability of attaining pCR.

The currently reported methods for predicting pCR after NAC include histomorphological analysis^11^, molecular biomarker analysis^12,13^, medical imaging tests^14,15^, and gene expression analysis^16,17^. However, these methods are expensive, complicated to operate, show low repeatability, and have certain limitations in clinical applications. Hence, quick, accurate, convenient, and economical prediction of pCR acquisition rate in patients after NAC remains a crucial problem in clinical treatment. In recent years, researchers have tried to use common serological markers to predict the pCR rate of breast cancer after NAC^18,19^. For example, one study confirmed that carbohydrate antigen 153(CA153) level before NAC in breast cancer patients was an independent predictor of pCR^20^. In these studies, the selected experimental indicators are conventional, simple, and economical, but the selected laboratory indicators are single and the accuracy is thus not high. Few authors have comprehensively studied which of the common laboratory test indicators can predict the pCR rate of breast cancer patients after receiving NAC; however, the prediction effect of these indicators combined are yet to be elucidated.

The nomogram is a prediction model that can intuitively predict the probability of an event, and is currently widely used in the prediction of clinical efficacy of various diseases^21^. The purpose of this study was to screen the laboratory indices related to the pCR rate of the primary site of breast cancer after NAC, and to establish the prediction model of the nomogram in order to provide a basis for clinicians to formulate more accurate treatment strategies for breast cancer.

## Results

### General pathological characteristics of patients and the relationship with bpCR rate after NAC

A total of 130 eligible patients (mean age: 47.5 years) were included in this study. Patients with cTNM stage II and stage III accounted for 76.9% (100/130) and 23.1% (30/130), respectively. The optimal cut-off value of Ki-67 was 65%, and the optimal cut-off value of BMI was 21.28 kg/m^2^. The detailed clinical and pathological characteristics of all 130 patients were presented in Table 1. Among these clinical characteristics, clinical stage, Ki-67 expression level, and BMI correlated with bpCR rate after NAC. Patients with low stage, high Ki-67, and low BMI index were more likely to reach bpCR after NAC. There was no significant statistical association between age, cT stage, cN stage, chemotherapy cycle, chemotherapy regimen, menstrual status, molecular typing, ER, PR, HER-2 expression, and bpCR rate.

**Table 1:** Clinical characteristics of 130 breast cancer patients and their association with bpCR rate after NAC.

Factors	Total	bpCR	Non-bpCR	P value	Factors	Total	bpCR	Non-bpCR	P value
	N(%)	N(%)	N(%)			N(%)	N(%)	N(%)	
Number of patients	130(100.0)	46(35.4)	84(64.6)		Menopausal status				0.304 *
Age (years, mean ± SD)	47.5 ± 10.3	46.2 ± 9.9	48.3 ± 10.5	0.234#	Premenopausal	77(59.2)	30(65.2)	47(56.0)	
**Clinical stage**				0.045*	Peri/postmenopausal	53(40.8)	16(34.8)	37(44.0)	
II	100(76.9)	40(87.0)	60(71.4)		Molecular subtype				0.676*
III	30(23.1)	6(13.0)	24(28.6)		Luminal B (Her2−)	9(6.9)	4(8.7)	5(6.0)	
**Clinical tumor stage**				0.598*	Luminal B (Her2 +)	42(32.3)	16(34.8)	26(31.0)	
cT0	1(0.8)	0(0.0)	1(1.2)		Her2 positive	57(43.8)	17(37.0)	40(47.6)	
cT1	15(11.5)	8(17.4)	7(8.3)		Triple negative	22(16.9)	9(19.6)	13(15.5)	
cT2	97(74.6)	32(69.6)	65(77.4)		Estrogen receptor				0.332*
cT3	14(10.8)	5(10.9)	9(10.7)		Negative	66(50.8)	26(56.5)	40(47.6)	
cT4	3(2.3)	1(2.2)	2(2.4)		Positive	64(49.2)	20(43.5)	44(52.4)	
**Clinical nodal stage**				0.262*	Progesterone receptor				0.324*
cN0	13(10.0)	6(13.0)	7(8.3)		Negative	92(70.8)	35(76.1)	57(67.9)	
cN1	90(69.2)	34(73.9)	56(66.7)		Positive	38(29.2)	11(23.9)	27(32.1)	
cN2	22(16.9)	4(8.7)	18(21.4)		HER-2				0.382*
cN3	5(3.8)	2(4.3)	3(3.6)		Negative	31(23.8)	13(28.3)	18(21.4)	
**Chemotherapy regimen**				0.591*	Positive	99(76.2)	33(71.7)	66(78.6)	
TCH	56(43.1)	22(47.8)	34(40.5)		Ki-67, %				0.033*
TEC	60(46.2)	20(43.5)	40(47.6)		< 65	98(76.0)	30(65.2)	68(81.9)	
EC-T	3(2.3)	0(0.0)	3(3.6)		≥ 65	31(24.0)	16(34.8)	15(18.1)	
TE	5(3.8)	1(2.2)	4(4.8)		Unknown	1			
TE + TP	1(0.8)	0(0.0)	1(1.2)		Body mass index, kg/m^2^				0.009*
TEC + TP	1(0.8)	1(2.2)	0(0.0)		< 21.28	31(23.8)	17(37.0)	14(16.7)	
THP	4(3.1)	2(4.3%)	2(2.4%)		≥ 21.28	99(76.2)	29(63.0)	70(83.3)	
**Chemotherapy cycle**				0.701*					
< 6	22(16.9)	7(15.2)	15(17.9)						
≥ 6	108(83.1)	39(84.8)	69(82.1)						

*NAC* neoadjuvant chemotherapy, *bpCR* breast pathological complete response, *TCH* docetaxel–caplurboplatin–trastuzumab, *TEC* docetaxel–epirubicin–cyclophosphamide, *EC-T* epirubicin–cyclophosphamide followed by docetaxel, *TE* docetaxel–epirubicin, *TP* docetaxel–cisplatin, *THP* docetaxel–trastuzumab–pertuzumab, *HER-2* human epidermal growth factor receptor 2.

*Chi-square test or Fisher exact test; ^#^Mann–Whitney U test.

### Association between common serological indicators and bpCR rate after NAC (univariable analysis)

**Figure 1:**
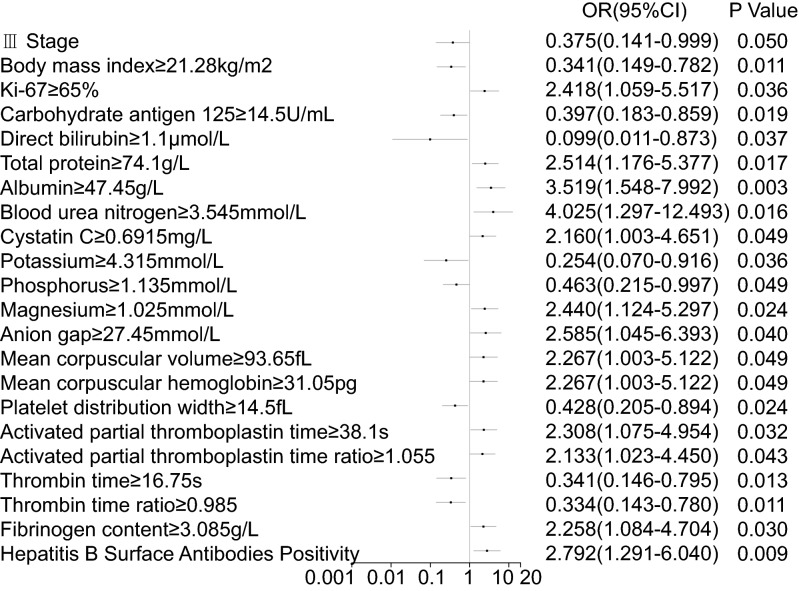
Single-factor logistic regression-based Forest plot analysis of the clinicopathological characteristics and serological indicators of 130 breast cancer patients related to breast pathologic complete response rate after neoadjuvant chemotherapy.

The association between bpCR and 65 indices was assessed (Supplementary Table S1 online). Among these, 19 indicators were correlated with the bpCR rate(p ≤ 0.05). The factors that showed positive association include total protein (TP), albumin (ALB), blood urea nitrogen (BUN), cystatin C (Cys-C), magnesium (Mg), anion gap (AG), mean corpuscular volume (MCV), mean corpuscular hemoglobin (MCH), activated partial thromboplastin time (APTT), activated partial thromboplastin time ratio (APTT R), fibrinogen content (FIB), and hepatitis B surface antibody positivity, whereas those that showed negative association include carbohydrate antigen 125(CA125), direct bilirubin (DAIL), potassium (K), phosphorus (POH), platelet distribution width (PDW), thrombin time (TT), and thrombin time ratio (TT R) (Table 2, Fig. 1).

**Table 2:** Serological indicators related to bpCR rate after NAC in 130 breast cancer patients.

Factors	Total	bpCR	Non-bpCR	P value	Factors	Total	bpCR	Non-bpCR	P value
	N(%)	N(%)	N(%)			N(%)	N(%)	N(%)	
Number of patients	130(100.0)	46(35.4)	84(64.6)		**Anion gap, mmol/L**				0.036
**Carbohydrate antigen 125, U/mL**				0.018	< 27.45	102(81.0)	32(71.1)	70(86.4)	
< 14.5	53(44.9)	25(59.5)	28(36.8)		≥ 27.45	24(19.0)	13(28.9)	11(13.6)	
≥ 14.5	65(55.1)	17(40.5)	48(63.2)		Unknown	4			
Unknown	12				**Complete blood count**				
**Liver function test**					**Mean corpuscular volume, fL**				0.046
**Direct bilirubin, μmol/L**				0.021	< 93.65	98(75.4)	30(65.2)	68(81.0)	
< 1.1	6(4.6)	5(10.9)	1(1.2)		≥ 93.65	32(24.6)	16(34.8)	16(19.0)	
≥ 1.1	124(95.4)	41(89.1)	83(98.8)		**Mean corpuscular hemoglobin, pg**				0.046
**Total protein, g/L**				0.016	< 31.05	98(75.4)	30(65.2)	68(81.0)	
< 74.15	58(44.6)	14(30.4)	44(52.4)		≥ 31.05	32(24.6)	16(34.8)	16(19.0)	
≥ 74.15	72(55.4)	32(69.6)	40(47.6)		**Platelet distribution width, fL**				0.023
**Albumin, g/L**				0.002	< 14.5	58(45.3)	27(58.7)	31(37.8)	
< 47.45	97(74.6)	27(58.7)	70(83.3)		≥ 14.5	70(54.7)	19(41.3)	51(62.2)	
≥ 47.45	33(25.4)	19(41.3)	14(16.7)		Unknown	2			
Kidney function test					**Coagulation function test**				
**Blood urea nitrogen, mmol/L**				0.011	**Activated partial thromboplastin time, s**				0.030
< 3.545	27(20.9)	4(8.7)	23(27.7)		< 38.1	89(68.5)	26(56.5)	63(75.0)	
≥ 3.545	102(79.1)	42(91.3)	60(72.3)		≥ 38.1	41(31.5)	20(43.5)	21(25.0)	
Unknown	1				**Activated partial thromboplastin time ratio**				0.042
**Cystatin C, mg/L**				0.047	< 1.055	66(51.2)	18(39.1)	48(57.8)	
< 0.6915	54(42.9)	14(31.1)	40(49.4)		≥ 1.055	63(48.8)	28(60.9)	35(42.2)	
≥ 0.6915	72(57.1)	31(68.9)	41(50.6)		Unknown	1			
Unknown	4				**Thrombin time, s**				0.011
Electrolyte test					< 16.75	86(66.2)	37(80.4)	49(58.3)	
**Potassium, mmol/L**				0.027	≥ 16.75	44(33.8)	9(19.6)	35(41.7)	
< 4.315	106(83.5)	42(93.3)	64(78.0)		**Thrombin time ratio**				0.009
≥ 4.315	21(16.5)	3(6.7)	18(22.0)		< 0.985	85(65.4)	37(80.4)	48(57.8)	
Unknown	3				≥ 0.985	44(33.8)	9(19.6)	35(42.2)	
**Phosphorus, mmol/L**				0.047	Unknown	1			
< 1.135	72(57.1)	31(68.9)	41(50.6)		**Fibrinogen content, g/L**				0.028
≥ 1.135	54(42.9)	14(31.1)	40(49.4)		< 3.085	76(58.5)	21(45.7)	55(65.5)	
Unknown	4				≥ 3.085	54(41.5)	25(54.3)	29(34.5)	
**Magnesium, mmol/L**				0.022	**Antibody of Hepatitis B surface**				0.008
< 1.025	86(68.3)	25(55.6)	61(75.3)		Negative	57(43.8)	13(28.3)	44(52.4)	
≥ 1.025	40(31.7)	20(44.4)	20(24.7)		Positive	73(56.2)	33(71.7)	40(47.6)	
Unknown	4								

*NAC* neoadjuvant chemotherapy, *bpCR* breast pathological complete response.

### Association between clinicopathological characteristics and common serological indicators and bpCR rate after NAC (multivariable logistic regression analysis)

Multivariable analysis was performed on the indicators included in the univariable analysis. Multivariable logistic regression showed that BMI, CA125, TP, BUN, Cys-C, K, POH, PDW, APTT, TT, and hepatitis B surface antibodies were independent predictors (Table 3). Of these factors, TP, BUN, Cys-C, APTT, and hepatitis B surface antibody positivity showed positive association to bpCR, while BMI, CA125, K, POH, PDW, and TT showed negative association.

**Table 3:** Multivariable logistic regression to identify predictors of bpCR after NAC based on clinicopathological characteristics and serological indicators.

Factors	Multivariable analysis		
	OR	95% CI	p value
**Body mass index, kg/m**^**2**^			
< 21.28	1		
≥ 21.28	0.082	0.012–0.559	0.011
Tumor marker test			
**Carbohydrate antigen 125, U/mL**			
< 14.5	1		
≥ 14.5	0.093	0.021–0.416	0.002
Liver function test			
**Total protein, g/L**			
< 74.15	1		
≥ 74.15	14.207	2.951–66.675	0.001
Kidney function test			
**Blood urea nitrogen, mmol/L**			
< 3.545	1		
≥ 3.545	39.512	2.775–562.500	0.007
**Cystatin C, mg/L**			
< 0.6915	1		
≥ 0.6915	8.363	1.859–37.609	0.006
Electrolyte test			
**Potassium, mmol/L**			
< 4.315	1		
≥ 4.315	0.116	0.014–0.983	0.048
**Phosphorus, mmol/L**			
< 1.135	1		
≥ 1.135	0.063	0.100–0.382	0.003
**Platelet distribution width, fL**			
< 14.5	1		
≥ 14.5	0.038	0.006–0.228	< 0.001
**Coagulation function test**			
**Activated partial thromboplastin time, s**			
< 38.1	1		
≥ 38.1	5.689	1.222–26.475	0.027
**Thrombin time, s**			
< 16.75	1		
≥ 16.75	0.145	0.032–0.657	0.012
**Antibody of hepatitis B surface**			
Negative	1		
Positive	4.899	1.183–20.214	0.028

*NAC* neoadjuvant chemotherapy, *bpCR* breast pathological complete response.

### Establishment and verification of the nomogram multi-factor prediction model

**Figure 2:**
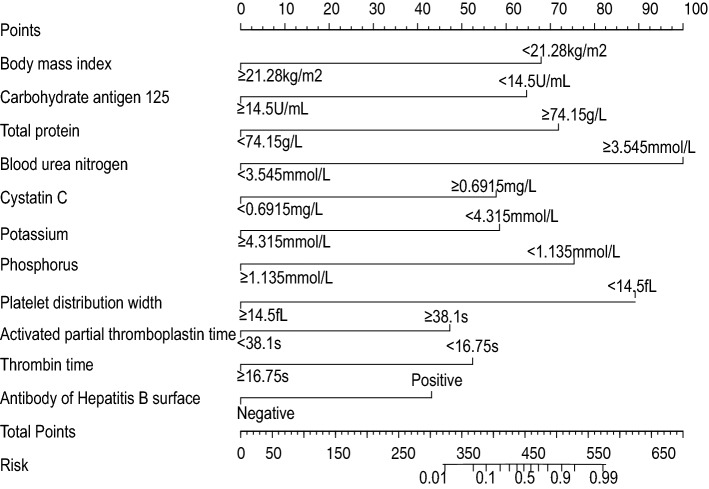
Nomogram to predict breast pathologic complete response rate of breast cancer patients after neoadjuvant chemotherapy based on clinicopathological characteristics and serological indices.

**Figure 3:**
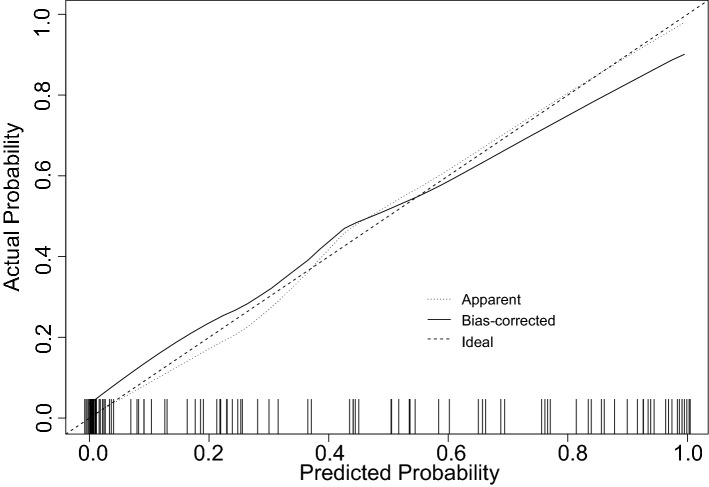
1000-bootstrap resampling internal verification correction for a nomogram of breast pathologic complete response rates after neoadjuvant chemotherapy in breast cancer patients with multiple factors.

**Figure 4:**
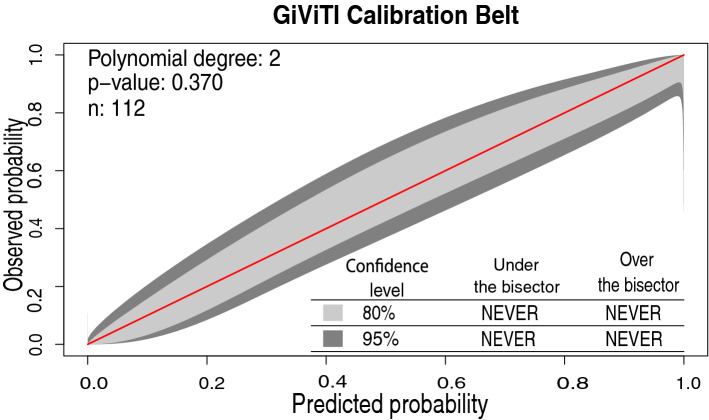
The GiViTI calibration belt for the nomogram of predicting the breast pathologic complete response rate of breast cancer patients after neoadjuvant chemotherapy.

**Figure 5:**
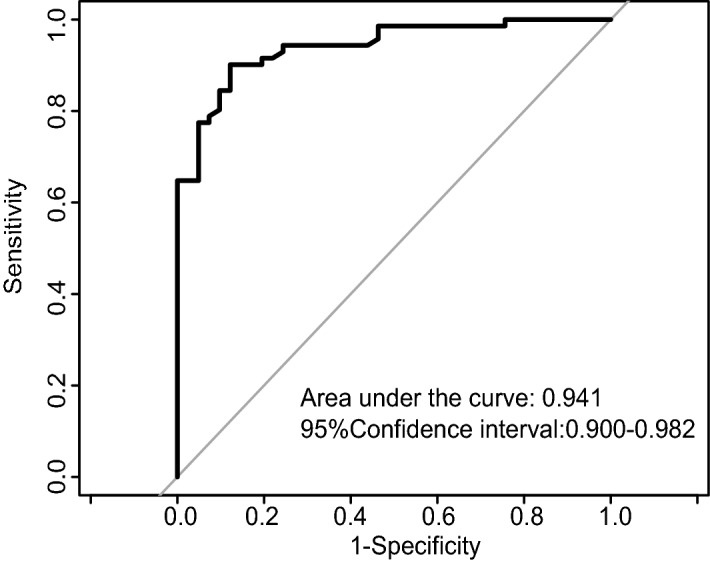
The receiver operating characteristic curve for a multivariable predictive breast pathologic complete response rate after neoadjuvant chemotherapy in breast cancer patients.

According to 11 clinical factors including BMI, CA125, TP, BUN, Cys-C, K, POH, PDW, APTT, TT, and hepatitis B surface antibody, a nomogram (Fig. 2) was developed to predict the bpCR rate of breast cancer patients after receipt of NAC. The predicted rate of bpCR can be obtained by summing the scores of the 11 factors. The Brier score of 0.095 indicated that the nomogram had a high accuracy. In the 1000 Bootstrap resampling internal verification calibration curve (Fig. 3), the trend of the predicted value and the true value were both consistent, and average absolute error between the predicted value and the true value was 0.041, indicating a good calibration between the predicted and actual observed values. The 95% CI of GiViTI calibration band (Fig. 4) did not cross the diagonal bisector, and the p value was 0.370, such that the nomogram was deemed as well calibrated. In the ROC curve analysis, the AUC of the nomogram was 0.941 (95% confidence interval: 0.900–0.982, Fig. 5). When the prediction probability was 0.560, the maximum value of Youden index was obtained, and at this time, the sensitivity was 90.1%; the specificity was 87.8%; all of which indicated that the nomogram had good discrimination and good prediction ability.

## Discussion

For breast cancer patients receiving NAC, predicting the probability of attaining bpCR before receiving NAC will help clinicians to formulate the most accurate treatment plan, thereby improving the prognosis. Laboratory-based markers have begun to play an increasingly important role in predicting pCR after NAC^18–20^. Laboratory indicators can be easily assessed and comprehensively reflect the patient's whole-body multi-system condition. However, previous studies only selected single laboratory indices, thereby resulting in relatively low prediction efficiency. In order to make the prediction more accurate, we need multi-system serum markers to fully predict the bpCR rate after NAC to achieve accurate individualized treatment for breast cancer patients.

A previous study found that a lower BMI before breast cancer diagnosis was associated with attaining pCR in NAC^22^; our results also confirmed this. BMI and breast cancer prognosis likely affect each other through metabolic factors^23,24^. The tumor marker CA125 is a prognostic marker for various tumors, especially gynecological tumors^25–27^. Among the three common breast cancer serum tumor markers—carcino-embryonic antigen(CEA), CA153, and CA125—we found that only CA125 was an independent predictor. Therefore, CA125 might be more valuable than CEA and CA153 in predicting the possibility of attaining bpCR after NAC in breast cancer.

Of the 13 tested indicators that reflected liver function, we found that only TB was an independent predictor. In patients with cancer, malnutrition can lead to many undesirable consequences such as decreased quality of life, decreased treatment response, and increased treatment-related toxicity^28^. TB is the main indicator of the nutritional status of the body. This study found that breast cancer patients with TB > 74.15 g/L were more likely to attain bpCR. In some reports, ALB levels could affect the long-term prognosis of breast cancer patients^29^ and high DBIL reduced the risk of breast cancer^30^; however in our study, while ALB and DBIL levels were related to the rate of obtaining bpCR, they are not independent predictors.

Of the five indicators reflecting renal function, only BUN and Cys-C were independent predictors. Studies have shown that elevated plasma Cys-C levels are observed in 40% of breast cancer patients and are positively correlated with tumor volume. Therefore, Cys-C can be used as a marker of breast cancer occurrence and progression^31^. Interestingly, in our study, patients with high levels of Cys-C were more likely to reach bpCR. The mechanism may be that Cys-C as a cysteine protease inhibitor can reduce cancer invasion and metastasis. This phenomenon was confirmed in ovarian cancer^32^, whether breast cancer has the same effect needs further research. In addition, we found that patients with low levels of BUN were more difficult to achieve bpCR. Urea is produced by the liver and excreted by the kidneys. Studies have shown that low levels of BUN suggested early damage to liver function^33^, and liver dysfunction is a risk factor for multiple tumor prognosis^34^.

In serum electrolyte testing, we found that low K, low POH, and high Mg were positively related factors of bpCR, while both K and POH levels were independent risk factors. According to research, increased serum potassium levels can be found in many types of tumors^35,36^. The likely mechanism of high potassium in serum promoting tumorigenesis and progression is as follows: (1) Higher levels of serum potassium itself can promote tumor growth through immune mechanisms^37^. For example, a cervical cancer study has shown that potassium inhibits the activity of T cells, leading to a decrease in the body's ability to prevent tumor progression^38^. (2) There may be some inherent individual factors that help regulate serum potassium, which also helps tumor progression. For example, genetic variations in ion channels and ion pumps involved in potassium homeostasis are associated with genes that regulate cell proliferation and differentiation^39,40^. Serum phosphate is an essential nutrient for the synthesis of nucleic acids, phospholipids, and high-energy metabolites such as adenosine triphosphate (ATP); thus, rapidly dividing cells require a continuous supply of phosphate^41,42^. High phosphorus in the serum of breast cancer patients may indicate the rapid proliferation of cancer cells. This is possibly why breast cancer patients with high serum phosphorus have a low bpCR rate.

PDW is an independent predictor in the complete blood count test. Various pro-inflammatory cytokines such as tumor necrosis factor-α, interleukin-1, and interleukin-6 are up-regulated as tumors develop and progress^43^. These cytokines promote the maturation of heterologous megakaryocytes, leading to the production and release of immature platelets of various characteristics and sizes into the circulatory system^44^, thereby increasing PDW values. This explains why breast cancer patients with high PDW have a worse prognosis^45^. Similar results were found in this study, and it is therefore more difficult for patients with high PDW to reach bpCR.

Tumor biology is closely related to coagulation^46^. Existing research showed that patients with malignant tumors often suffered from coagulation dysfunction, which manifested as activation of the coagulation system and fibrinolytic system^47,48^. In this study, two coagulation indicators—APTT and TT—were independently related to bpCR after NAC in breast cancer patients. Patients with low APTT and high TT have lower bpCR rates. Abnormal coagulation function not only increases the possibility of thromboembolism, but also promotes tumor growth and the spread of cancer cells^49^, thus affecting the tumor's response to treatment.

In this study, we found that breast cancer patients with hepatitis B surface antibody positivity were more likely to reach bpCR. The mechanism of hepatitis B virus (HBV) and breast cancer progression and prognosis is unclear, but reports have shown that HBV infection is a risk factor for breast cancer^50^. The mechanism may be related to the interference of hepatitis B virus X protein (HBx) on cell repair mechanism, which leads to cell carcinogenesis^51^. In addition, HBV infection causes liver dysfunction, which affects estrogen regulation, and estrogen is a known risk factor for breast cancer. Therefore, HBV infection may indirectly induce breast epithelial cells to become cancerous^50^.

The human body is a multi-system coordinated and integrated organic entity. Tumors not only affect the local organs and tissues but also the functions of various systems throughout the body. Further, the function of each system in the whole body also reflects the existing degree of tumor influence and the future prognosis of the disease to some extent. Therefore, laboratory indicators can reveal disease prognosis to a certain extent. We analyzed several clinical factors and 65 common laboratory indicators, including tumor markers, liver function indicators, renal function indicators, electrolyte test indicators, complete blood count test indicators, coagulation function tests indicators, and hepatitis B surface antibody to analyze the association between these indicators and bpCR. The selected indicators comprehensively covered the clinical characteristics of tumors, and considered the impact of patients' multiple system functions on bpCR. Finally, 11 independent predictors were selected, and a nomogram model for predicting bpCR after NAC in breast cancer patients was successfully established. The AUC of the ROC and the calibration curve indicated that nomogram had good discrimination and calibration. The Brier score showed that the nomogram had high prediction accuracy. These results confirmed that our nomogram had high predictive value. We believe our model can help clinicians to accurately predict the response of breast cancer patients to NAC and the possibility of attaining bpCR, and select and formulate the most beneficial individualized treatment plan for patients in a timely manner. In addition, our model is also economically advantageous, because we choose conventional clinical pathological characteristics and common serological laboratory indicators, which will not increase the financial burden of patients.

Our study has some limitations. First, this is a retrospective analysis performed in a single -center, and the number of patients involved is relatively small. In this study, many factors were tested, so there existed the risk of overfitting. Therefore, large-scale, multicenter studies are necessary to further validate our findings and the accuracy of the nomogram. Second, there are unknown factors related to bpCR that we have not yet evaluated. For example, tumor staging, hormone receptors, HER-2, chemotherapeutic and endocrine therapy drugs, Ki-67, and molecular typing have been shown to be related to breast cancer prognosis in some reports^52–55^, but our research has failed to confirm that these factors are related to bpCR or independent predictors of bpCR. The specific mechanism needs to be further explored. Third, we used binary variables instead of continuous variables to study their relationship with bpCR. Therefore, dichotomy of these biomarkers may lead to the loss of information and the loss of nomogram performance. In addition, there are some imbalances in the clinical characteristics of the study population. For example, the proportion of Her2-positive patients was very high, while there were no Luminal A patients. The possible reason is that according to NCCN Breast Cancer Clinical Practice Guidelines, pCR of Luminal A patients does not provide survival benefits. Therefore, we generally do not recommend NAC for Luminal A patients unless the tumor is too large to be resected, which is also the reason why there is no Luminal A in our cases. Whether our findings are also applicable to Luminal A patients needs further study and discussion.

In Conclusion, we screened 11 independent predictors related to bpCR after NAC from routine clinicopathological features and laboratory serum markers of breast cancer, and successfully constructed a nomogram prediction model with accurate and specific predictions, which do not increase the economic burden of patients, and have high clinical application value.

## Methods

### Patients and factors

**Figure 6:**
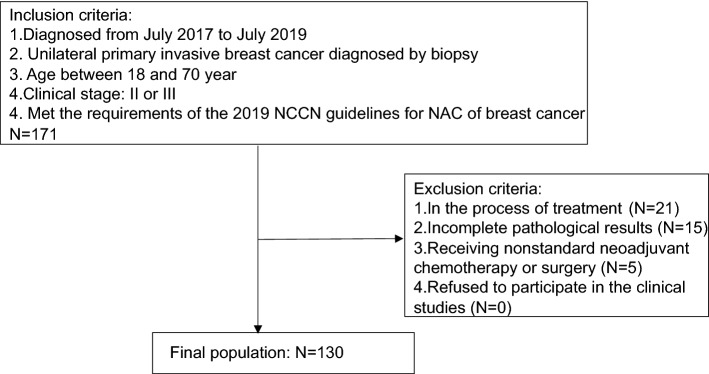
Eligibility, inclusion, and exclusion criteria of study population.

In this retrospective study, patients who were treated in the breast surgery department of the First Affiliated Hospital of Xi'an Jiaotong University from July 2017 to July 2019 were enrolled. The inclusion criteria were as follows: (1) unilateral primary invasive breast cancer diagnosed by biopsy; (2) age between 18 and 70 years; (3) clinical stage: II or III; (4) met the requirements of the 2019 National Comprehensive Cancer Network (NCCN) guidelines for NAC of breast cancer^56^; The following patients were excluded: (1) those in the process of treatment; (2) those with incomplete pathological results; (3) those that did not receive standardized chemotherapy or surgical procedures; and (4) those who refused to participate in the relevant clinical studies. Finally, 130 patients were included. The detailed screening process is shown in Fig. 6. The pathological and clinical data of all patients were obtained from the electronic medical records separately by two independent data collectors, and were checked by a third data collector. The testing for all clinical factors and laboratory indicators were completed within three days before the patients received NAC.

The common clinical factors included age, clinical stage, clinical tumor stage, clinical lymph node stage, chemotherapy regimen, chemotherapy cycle, menstrual status, molecular typing, estrogen receptor (ER), progesterone receptor (PR), Human epidermal growth factor receptor 2 (HER-2), Ki-67, and body mass index (BMI). The laboratory indicators included tumor markers, liver function indicators, renal function indicators, electrolyte detection indicators, complete blood count indicators, coagulation function tests indicators, and hepatitis B surface antibody detection, 65 indicators in total.

All patients received chemotherapy based on taxane and/or anthracyclines in the NAC. Trastuzumab was added to Her2-positive patients’ treatment regimen, while some patients received pertuzumab. The tumor size was evaluated by physical examination and B-ultrasound in each cycle, and was evaluated by MRI in every two cycles. According to the NCCN guideline evaluation criteria, those who were effective should complete the neoadjuvant chemotherapy according to the established plan and cycle. When the tumor was not relieved, the treatment plan and cycle should be adjusted in time, and those who were still ineffective after adjustment should be operated. The formulation of the treatment plan for all patients was determined in accordance with the 2019 version of the NCCN Breast Cancer Clinical Practice Guidelines^56^ and discussed by general practitioners.

The clinical stage was determined according to the 8th edition of the clinical stage of breast cancer recommended by the American Joint Committee on Cancer (AJCC)^57^. By immunohistochemical staining, ER and PR expression levels < 1% were considered negative. HER-2 status was determined according to the American Society of Clinical Oncology/College of American Pathologists (ASCO/CAP) guidelines^58^. Molecular typing was determined by St Gallen guidelines^59^. According to the NCCN guidelines^56^, the complete response of the primary pathological site of the breast was defined as histological evidence that no invasive tumor was found in the primary breast lesions after NAC, regardless of the presence of residual ductal carcinoma in situ, namely bpCR(ypT0/is).

BMI is calculated by dividing the patient's weight (kg) by the square of height (m^2^), that is, BMI = weight (kg)/height^2^ (m^2^). Hepatitis B surface antibody < 10 mIU/mL as negative and ≥ 10 mIU/mL was positive.

Each patient provided informed consent before treatment. The Study was conducted in accordance with the Declaration of Helsinki and the research code of the First Affiliated Hospital of Xi'an Jiaotong University. And the study was approved by the Ethics Committee of the First Affiliated Hospital of Xi'an Jiaotong University.

Complete blood count was conducted by blood analyzer BC5390 (Mindray, Shenzhen, China); coagulation function test was conducted by automatic hemagglutination apparatus CA7000 (Sysmex, Kobe, Japan); liver function, renal function, and electrolyte tests were conducted by automatic biochemical immune analyzer VITROS5600 (JNJ, New Jersey, US) ; Hepatitis B surface antibody test was conducted by automatic immune analyzer I2000SR (Abbott, Illinois, US).

### Statistical analysis

Of all clinical factors and laboratory indicators, only age was analyzed as a continuous variable, while all other indicators were analyzed as categorical variables. Continuous variables other than age were divided into two groups according to the optimal cut-off value, which was determined by calculating the maximum Youden index (sensitivity + specificity − 1) from the receiver operating characteristic (ROC) curve^60^. The chi-square test or Mann–Whitney U test was used to analyze the association between bpCR and the index. If the expected frequency was < 5, Fisher's exact test was used. All p values were two-sided. Indexes with p ≤ 0.05 in the chi-square test, Mann–Whitney U test, or Fisher’s exact test were included in forward stepwise logistic regression (likelihood ratio). Forest plots were drawn based on the results of the univariable analysis, and multivariable binary logistic regression was used to determine independent predictors of bpCR after NAC. Then, based on the multivariable logistic regression model, the nomogram was established. Calibration of the nomogram was carried out by the 1000 bootstrap resampling internal verification and was displayed by the calibration curve. The agreement between the predicted and observed probability was shown by the GiViTI calibration band. The Brier score was calculated to measure the prediction accuracy. The ROC curve was used to display the nomogram discrimination, and the discrimination was quantified by area under the curve (AUC) and the concordance index (C-index). Statistical analysis was performed the IBM SPSS Statistics version 22.0 (IBM Corporation, Armonk, NY, USA) and R software (version 3.6.2; The R Foundation for Statistical Computing, Austria, Vienna). For all analyses, p ≤ 0.05 was considered to indicate statistical significance.

## Supplementary Information

Supplementary Table 1.

## Data Availability

The datasets analysed during the current study are available from the corresponding author on reasonable request.
