# Supplementary material for: A nomogram based on clinicopathological features and serological indicators predicting breast pathologic complete response of neoadjuvant chemotherapy in breast cancer
Source: Sci Rep. 2021 May 31;11:11348. doi: 10.1038/s41598-021-91049-x (PMC8167133; doi:10.1038/s41598-021-91049-x)
Supplement: Supplementary file 1 — Supplementary Table 1. [file 41598_2021_91049_MOESM1_ESM.docx]

**A** **Nomogram** **Based on** **Clinicopathological Features and Serological Indicators Predicting** **Breast Pathologic Complete Response of Neoadjuvant Chemotherapy in Breast Cancer**

**Yijun Li^1^, Jian Zhang^1^, Bin Wang^1^, Huimin Zhang^1^, Jianjun He^1*^, Ke Wang^1*^**

^1^Departments of Breast Surgery, First Affiliate Hospital of Xi’an Jiaotong University, Xi’an, People’s Republic of China;

***** **Co-corresponding author:**

Jianjun He, M.D., Department of Breast Surgery, First Affiliated Hospital of Xi'an Jiaotong University.Address:277 Yanta West Road, Xi'an 710061, China. Telephone: 86-13992808259; Fax: 86-029-85324605; E-mail: chinahjj@163.com

****Corresponding author:**

Ke Wang, M.D., Department of Breast Surgery, First Affiliated Hospital of Xi'an Jiaotong University.Address:277 Yanta West Road, Xi'an 710061, China. Telephone:86-18991232545; Fax:86-029-85324605; E-mail: 979915080@qq.com

Supplementary Table 1: Serological indicators of 130 breast cancer patients and their correlation with bpCR rate after NAC

| Factors | Total | bpCR r | Non-bpCR | P value |  |
| --- | --- | --- | --- | --- | --- |
|  | N(%) | N(%) | N(%) |  |  |
| Number of patients | 130(100.0) | 46(35.4) | 84(64.6) |  |  |
| Carcino-embryonic antigen, ng/mL | |  |  | 0.081 |  |
| <2.025 | 66(55.9) | 28(66.7) | 38(50.0) |  |  |
| ≥2.025 | 52(44.1) | 14(33.3) | 38(50.0) |  |  |
| Unknown | 12 |  |  |  |  |
| Carbohydrate antigen125,U/mL | |  |  | **0.018** |  |
| <14.5 | 53(44.9) | 25(59.5) | 28(36.8) |  |  |
| ≥14.5 | 65(55.1) | 17(40.5) | 48(63.2) |  |  |
| Unknown | 12 |  |  |  |  |
| Carbohydrate antigen 153,U/mL | |  |  | 0.218 |  |
| <7.53 | 13(11.0) | 7(16.7) | 6(7.9) |  |  |
| ≥7.53 | 105(89.0) | 35(83.3) | 70(92.1) |  |  |
| Unknown | 12 |  |  |  |  |
| **Liver function test** |  |  |  |  |  |
| Aspartate transaminase, U/L |  |  |  | 0.130 |  |
| <16.5 | 42(32.3) | 11(23.9) | 31(36.9) |  |  |
| ≥16.5 | 88(67.7) | 35(76.1) | 53(63.1) |  |  |
| Alanine transaminase ,U/L |  |  |  | 0.206 |  |
| <22.5 | 96(73.8) | 37(80.4) | 59(70.2) |  |  |
| ≥22.5 | 34(26.2) | 9(19.6) | 25(29.8) |  |  |
| Aspartate transaminase/Alanine transaminase | |  |  | 0.113 |  |
| <1.25 | 77(59.2) | 23(50.0) | 54(64.3) |  |  |
| ≥1.25 | 53(40.8) | 23(50.0) | 30(35.7) |  |  |
| Alkaline phosphatase, U/L |  |  |  | 0.166 |  |
| <82.5 | 81(62.3) | 25(54.3) | 56(66.7) |  |  |
| ≥82.5 | 49(37.7) | 21(45.7) | 28(33.3) |  |  |
| Gamma-glutamyl transpeptidase, U/L | |  |  | 0.064 |  |
| <26 | 108(83.1) | 42(91.3) | 66(78.6) |  |  |
| ≥26 | 22(16.9) | 4(8.7) | 18(21.4) |  |  |
| Total cholesterol, mmol/L |  |  |  | 0.130 |  |
| <4.875 | 88(67.7) | 35(76.1) | 53(63.1) |  |  |
| ≥4.875 | 42(32.3) | 11(23.9) | 31(36.9) |  |  |
| Total bilirubin, μmol/L |  |  |  | 0.206 |  |
| <14.25 | 81(62.3) | 32(69.6) | 49(58.3) |  |  |
| ≥14.25 | 49(37.7) | 14(30.4) | 35(41.7) |  |  |
| Direct bilirubin, μmol/L |  |  |  | **0.021** |  |
| <1.1 | 6(4.6) | 5(10.9) | 1(1.2) |  |  |
| ≥1.1 | 124(95.4) | 41(89.1) | 83(98.8) |  |  |
| Indirect bilirubin, μmol/L |  |  |  | 0.199 |  |
| <9.2 | 75(57.7) | 30(65.2) | 45(53.6) |  |  |
| ≥9.2 | 55(42.3) | 16（34.8） | 39(46.4) |  |  |
| Total protein, g/L |  |  |  | **0.016** |  |
| <74.15 | 58(44.6) | 14(30.4) | 44(52.4) |  |  |
| ≥74.15 | 72(55.4) | 32(69.6) | 40(47.6) |  |  |
| Albumin, g/L |  |  |  | **0.002** |  |
| <47.45 | 97(74.6) | 27(58.7) | 70(83.3) |  |  |
| ≥47.45 | 33(25.4) | 19(41.3) | 14(16.7) |  |  |
| Globulin, g/L |  |  |  | 0.129 |  |
| <28.15 | 59(45.4) | 25(54.3) | 34(40.5) |  |  |
| ≥28.15 | 71(54.6) | 21(45.7) | 50(59.5) |  |  |
| Albumin/Globulin |  |  |  | 0.271 |  |
| <1.55 | 65(50.0) | 20(43.5) | 45(53.6) |  |  |
| ≥1.55 | 65(50.0) | 26(56.5) | 39(46.4) |  |  |
| **Kidney function test** |  |  |  |  |  |
| Blood Urea nitrogen,mmol/L |  |  |  | **0.011** |  |
| <3.545 | 27(20.9) | 4(8.7) | 23(27.7) |  |  |
| ≥3.545 | 102(79.1) | 42(91.3) | 60(72.3) |  |  |
| Unknown | 1 |  |  |  |  |
| Creatinine, μmol/L |  |  |  | 0.141 |  |
| <43.5 | 29(22.5) | 7(15.2) | 22(26.5) |  |  |
| ≥43.5 | 100(77.5) | 39(84.8) | 61(73.5) |  |  |
| Unknown | 1 |  |  |  |  |
| Cystatin C, mg/L |  |  |  | **0.047** |  |
| <0.6915 | 54(42.9) | 14(31.1) | 40(49.4) |  |  |
| ≥0.6915 | 72(57.1) | 31(68.9) | 41(50.6) |  |  |
| Unknown | 4( |  |  |  |  |
| Glucose, mmol/L |  |  |  | 0.337 |  |
| <5.005 | 69(53.5) | 22(47.8) | 47(56.6) |  |  |
| ≥5.005 | 60(46.5) | 24(52.2) | 36(43.4) |  |  |
| Unknown | 1 |  |  |  |  |
| Uric Acid, μmol/L |  |  |  | 0.149 |  |
| <243.5 | 50(38.8) | 14(30.4) | 36(43.4) |  |  |
| ≥243.5 | 79(61.2) | 32(69.6) | 47(56.6) |  |  |
| Unknown | 1 |  |  |  |  |
| **Electrolyte test** |  |  |  |  |  |
| Potassium, mmol/L |  |  |  | **0.027** |  |
| <4.315 | 106(83.5) | 42(93.3) | 64(78.0) |  |  |
| ≥4.315 | 21(16.5) | 3(6.7) | 18(22.0) |  |  |
| Unknown | 3 |  |  |  |  |
| Sodium, mmol/L |  |  |  | 0.085 |  |
| <140.5 | 31(24.4) | 7(15.6) | 24(29.3) |  |  |
| ≥140.5 | 96(75.6) | 38(84.4) | 58(70.7) |  |  |
| Unknown | 3 |  |  |  |  |
| Chlorine, mmol/L |  |  |  | 0.268 |  |
| <101.95 | 51(40.2) | 21(46.7) | 30(36.6) |  |  |
| ≥101.95 | 76(59.8) | 24(53.3) | 52(63.4) |  |  |
| Unknown | 3 |  |  |  |  |
| Phosphorus, mmol/L |  |  |  | **0.047** |  |
| <1.135 | 72(57.1) | 31(68.9) | 41(50.6) |  |  |
| ≥1.135 | 54(42.9) | 14(31.1) | 40(49.4) |  |  |
| Unknown | 4 |  |  |  |  |
| Calcium, mmol/L |  |  |  | 0.156 |  |
| <2.315 | 70(55.1) | 21(46.7) | 49(59.8) |  |  |
| ≥2.315 | 57(44.9) | 24(53.3) | 33(40.2) |  |  |
| Unknown | 3 |  |  |  |  |
| Magnesium, mmol/L |  |  |  | **0.022** |  |
| <1.025 | 86(68.3) | 25(55.6) | 61(75.3) |  |  |
| ≥1.025 | 40(31.7) | 20(44.4) | 20(24.7) |  |  |
| Unknown | 4 |  |  |  |  |
| Carbon dioxide binding rate, mmol/L | |  |  | 0.226 |  |
| <23.25 | 67(52.8) | 27(60.0) | 40(48.8) |  |  |
| ≥23.25 | 60(47.2) | 18(40.0) | 42(51.2) |  |  |
| Unknown | 3 |  |  |  |  |
| Anion gap, mmol/L |  |  |  | **0.036** |  |
| <27.45 | 102(81.0) | 32(71.1) | 70(86.4) |  |  |
| ≥27.45 | 24(19.0) | 13(28.9) | 11(13.6) |  |  |
| Unknown | 4 |  |  |  |  |
| **Complete blood count** |  |  |  |  |  |
| Red blood cell count/L |  |  |  | 0.206 |  |
| <4.525 | 81(62.3) | 32(69.6) | 49(58.3) |  |  |
| ≥4.525 | 49(37.7) | 14(30.4) | 35(41.7) |  |  |
| Hemoglobin, g/L |  |  |  | 0.282 |  |
| <135.5 | 76(58.5) | 24(52.2) | 52(61.9) |  |  |
| ≥135.5 | 54(41.5) | 22(47.8) | 32(38.1) |  |  |
| Hematocrit,% |  |  |  | 0.153 |  |
| <41.2 | 84(64.6) | 26(56.55) | 58(69.0) |  |  |
| ≥41.2 | 46(35.4) | 20(43.5) | 26(31.0) |  |  |
| Mean corpuscular volume, fL | |  |  | **0.046** |  |
| <93.65 | 98(75.4) | 30(65.2) | 68(81.0) |  |  |
| ≥93.65 | 32(24.6) | 16(34.8) | 16(19.0) |  |  |
| Mean corpuscular hemoglobin, pg |  |  |  | **0.046** |  |
| <31.05 | 98(75.4) | 30(65.2) | 68(81.0) |  |  |
| ≥31.05 | 32(24.6) | 16(34.8) | 16(19.0) |  |  |
| Mean corpuscular hemoglobin concentration, g/L | | |  | 0.185 |  |
| <324.5 | 25(19.2) | 6(13.0) | 19(22.6) |  |  |
| ≥324.5 | 105(80.8) | 40(87.0) | 65(77.4) |  |  |
|  |  |  |  |  |  |
| Red blood cell distribution width-  Coefficient of variation,% | |  |  | 0.108 |  |
| <13.45 | 103(79.2) | 40(87.0) | 63(75.0) |  |  |
| ≥13.45 | 27(20.8) | 6(13.0) | 21(25.0) |  |  |
| Red blood cell distribution width-  Standard deviation,fL | |  |  | 0.257 |  |
| <43.25 | 82(63.1) | 32(69.6) | 50(59.5) |  |  |
| ≥43.25 | 48(36.0) | 14（30.4） | 34(40.5) |  |  |
| Platelet count,10^9/L |  |  |  | 0.185 |  |
| <299 | 105(80.8) | 40(87.0) | 65(77.4) |  |  |
| ≥299 | 25(19.2) | 6(13.0) | 19(22.6) |  |  |
| Platelet distribution width,fL |  |  |  | **0.023** |  |
| <14.5 | 58(45.3) | 27(58.7) | 31(37.8) |  |  |
| ≥14.5 | 70(54.7) | 19(41.3) | 51(62.2) |  |  |
| Unknown | 2 |  |  |  |  |
| Mean platelet volume,fL |  |  |  | 0.062 |  |
| <11.75 | 87(68.0) | 36(78.3) | 51(62.2) |  |  |
| ≥11.75 | 41(32.0) | 10(21.7) | 31(37.8) |  |  |
| Unknown | 2 |  |  |  |  |
| Platelet-large cell ratio,% |  |  |  | 0.107 |  |
| <38.45 | 83(64.8) | 34(73.9) | 49(59.8) |  |  |
| ≥38.45 | 45(35.2) | 12(26.1) | 33(40.0) |  |  |
| Unknown | 2 |  |  |  |  |
| Thrombocytocrit,% |  |  |  | 0.411 |  |
| <0.285 | 86(67.2) | 33(71.7) | 53(64.6) |  |  |
| ≥0.285 | 42(32.8) | 13(28.3) | 29(35.4) |  |  |
| Unknown | 2 |  |  |  |  |
| White blood cell count,10^9/L |  |  |  | 0.157 |  |
| <5.365 | 57(43.8) | 24(52.2) | 33(39.3) |  |  |
| ≥5.365 | 73(56.2) | 22(47.8) | 51(60.7) |  |  |
| Lymphocyte count,10^9/L |  |  |  | 0.127 |  |
| <1.325 | 48(36.9) | 21(45.7) | 27(32.1) |  |  |
| ≥1.325 | 82(63.1) | 25(54.3) | 57(67.9) |  |  |
| Monocyte count,10^9/L |  |  |  | 0.104 |  |
| <0.185 | 17(13.1) | 9(19.6) | 8(9.5) |  |  |
| ≥0.185 | 113(86.9) | 37(80.4) | 76(90.5) |  |  |
| Neutrophil count,10^9/L |  |  |  | 0.384 |  |
| <4.08 | 80(61.5) | 26(56.5) | 54(64.3) |  |  |
| ≥4.08 | 50(38.5) | 20(43.5) | 30(35.7) |  |  |
| Eosinophil count,10^9/L |  |  |  | 0.152 |  |
| <0.025 | 38(29.2) | 17(37.0) | 21(25.0) |  |  |
| ≥0.025 | 92(70.8) | 29(63.0) | 63(75.0) |  |  |
| Basophil count,10^9/L |  |  |  | 0.203 |  |
| <0.025 | 78(60.0) | 31(67.4) | 47(56.0) |  |  |
| ≥0.025 | 52(40.0) | 15(32.6) | 37(44.0) |  |  |
| Lymphocyte percentage,% |  |  |  | 0.218 |  |
| <23.1 | 42(32.3) | 18(39.1) | 24(28.6) |  |  |
| ≥23.1 | 88(67.7) | 28(60.9) | 60(71.4) |  |  |
| Monocyte percentage,% |  |  |  | 0.184 |  |
| <5.35 | 86(66.2) | 27(58.7) | 59(70.2) |  |  |
| ≥5.35 | 44(33.8) | 19(41.3) | 25(29.8) |  |  |
| Neutrophil percentage,% |  |  |  | 0.188 |  |
| <64.25 | 61(46.9) | 18(39.1) | 43(51.2) |  |  |
| ≥64.25 | 69(53.1) | 28(60.9) | 41(48.8) |  |  |
| Eosinophil percentage,% |  |  |  | 0.209 |  |
| <0.85 | 61(46.9) | 25(54.3) | 36(42.9) |  |  |
| ≥0.85 | 69(53.1) | 21(45.7) | 48(57.1) |  |  |
| Basophil percentage,% |  |  |  | 0.157 |  |
| <0.35 | 57(43.8) | 24(52.2) | 33(39.3) |  |  |
| ≥0.35 | 73(56.2) | 22(47.8) | 51(60.7) |  |  |
| **Coagulation function test** |  |  |  |  |  |
| Prothrombin time,S |  |  |  | 0.444 |  |
| <13.15 | 82(63.1) | 27(58.7) | 55(65.5) |  |  |
| ≥13.15 | 48(36.9) | 19(41.3) | 29(34.5) |  |  |
| Prothrombin activity,% |  |  |  | 0.126 |  |
| <93.3 | 40(30.8) | 18(39.1) | 22(26.2) |  |  |
| ≥93.3 | 90(69.2) | 28(60.9) | 62(73.8) |  |  |
| Prothrombin ratio |  |  |  | 0.104 |  |
| <1.075 | 113(86.9) | 37(80.4) | 76(90.5) |  |  |
| ≥1.075 | 17(13.1) | 9(19.6) | 8(9.5) |  |  |
| Prothrombin International normalized ratio | |  |  | 0.198 |  |
| <1.015 | 83(63.8) | 26(56.5) | 57(67.9) |  |  |
| ≥1.015 | 47(36.2) | 20(43.5) | 27(32.1) |  |  |
| Activated partial thromboplastin time,S | |  |  | **0.030** |  |
| <38.1 | 89(68.5) | 26(56.5) | 63(75.0) |  |  |
| ≥38.1 | 41(31.5) | 20(43.5) | 21(25.0) |  |  |
| Activated partial thromboplastin time ratio | |  |  | **0.042** |  |
| <1.055 | 66(51.2) | 18(39.1) | 48(57.8) |  |  |
| ≥1.055 | 63(48.8) | 28(60.9) | 35(42.2) |  |  |
| Unknown | 1 |  |  |  |  |
| Thrombin time,S |  |  |  | **0.011** |  |
| <16.75 | 86(66.2) | 37(80.4) | 49(58.3) |  |  |
| ≥16.75 | 44(33.8) | 9(19.6) | 35(41.7) |  |  |
| Thrombin time ratio |  |  |  | **0.009** |  |
| <0.985 | 85(65.4) | 37(80.4) | 48(57.8) |  |  |
| ≥0.985 | 44(33.8) | 9(19.6) | 35(42.2) |  |  |
| Unknown | 1 |  |  |  |  |
| Fibrinogen content, g/L |  |  |  | **0.028** |  |
| <3.085 | 76(58.5) | 21(45.7) | 55(65.5) |  |  |
| ≥3.085 | 54(41.5) | 25(54.3) | 29(34.5) |  |  |
| D-dimer, mg/L |  |  |  | 0.075 |  |
| <0.55 | 86(71.7) | 28(62.2) | 58(77.3) |  |  |
| ≥0.55 | 34(28.3) | 17(37.8) | 17(22.7) |  |  |
| Fibrinogen degradation products, mg/L | |  |  | 0.341 |  |
| <1.05 | 52(43.3) | 22(48.9) | 30(40.0) |  |  |
| ≥1.55 | 68(56.7) | 23(51.1) | 45(60.0) |  |  |
| Unknown | 10 |  |  |  |  |
| Antibody of Hepatitis B surface |  |  |  | **0.008** |  |
| Negative | 57(43.8) | 13(28.3) | 44(52.4) |  | |
| Positive | 73(56.2) | 33(71.7) | 40(47.6) |  | |

NAC: Neoadjuvant chemotherapy

bpCR: Breast pathological complete response
